# Muscle Oxygen Saturation Dynamics During Upper-Body Resistance Exercise

**DOI:** 10.3390/s24206668

**Published:** 2024-10-16

**Authors:** Adam M. Gonzalez, Gerald T. Mangine, Anthony G. Pinzone, Kyle S. Beyer, Jeremy R. Townsend

**Affiliations:** 1 Department of Allied Health and Kinesiology, Hofstra University, Hempstead, NY 11549, USA; 2 Department of Exercise Science and Sport Management, Kennesaw State University, Kennesaw, GA 30144, USA; gmangine@kennesaw.edu; 3 Program in Exercise Science and Exercise Physiology, Kent State University, Kent, OH 44240, USA; apinzone@kent.edu; 4 Resistance Exercise, Physiology, and Sport Laboratory, Health and Exercise Physiology Department, Ursinus College, Collegeville, PA 19426, USA; kbeyer@ursinus.edu; 5 Research, Nutrition and Innovation, AG1, Carson City, NV 89701, USA; jeremy.townsend@drinkag1.com

**Keywords:** near-infrared spectroscopy, bench press, muscle oxygenation, SmO_2_, muscle oxygen re-saturation

## Abstract

Research examining the changes in muscle oxygen saturation across multiple sets of resistance exercise is limited. The purpose of this study was to describe the physiological response of muscle oxygenation parameters during upper-body resistance exercise and examine the differential effects of relevant participant characteristics on resistance training performance and muscle oxygen saturation dynamics. Sixty-one recreationally trained men (n = 44; 21.8 ± 2.6 years) and women (n = 17; 20.2 ± 1.8 years) completed five-repetition maximum sets of barbell bench presses at a load equal to 75% 1-RM with a 2 min rest interval. Muscle oxygen saturation (SmO_2_) dynamics within the anterior deltoid were monitored using a portable near-infrared spectroscopy sensor. The percent change in SmO_2_ (∆%SmO_2_), the muscle oxygen re-saturation rate (SmO_2_RecSlope), and the highest measured SmO_2_ value during recovery periods (SmO_2_Peak) were measured. Two-way (sex [men, women] x time [sets 1–5]) repeated measures analyses of variance (ANOVA) were performed on muscle saturation variables. To examine the effect of relevant controlling variables, separate analyses of covariance (ANCOVA) with repeated measures were also performed. No differences were seen with ∆%SmO_2_ across sets. The main effects for sets occurred for SmO_2_RecSlope, whereby a decline was noted on sets 4 and 5 (*p* = 0.001) compared to set 1. Additionally, SmO_2_Peak was the lowest on set 5 (*p* < 0.001) compared to all other sets. Moreover, body mass (*p* = 0.013), diastolic blood pressure (*p* = 0.044), and mean arterial pressure (*p* = 0.033) for ∆%SmO_2_ were the only significant covariates noted amongst the muscle oxygenation variables. In conclusion, no sex differences and only a few set differences in muscle oxygen saturation dynamics were seen without employing any covariates. Body mass, diastolic blood pressure, and mean arterial pressure were identified as factors that could influence observed responses.

## 1. Introduction

Wireless near-infrared spectroscopy (NIRS) has emerged as a low-cost, non-invasive technology for real-time observation of muscle oxygenation–deoxygenation dynamics [1,2]. The technology is based on a modified Beer–Lambert Law, where infrared light is emitted into muscle capillaries (in the region over which the device is attached) to analyze the amount of light absorbed at wavelengths pertaining to oxygenated and deoxygenated hemoglobin [3,4]. Devices based on this technology have proved to be a reliable method for measuring localized blood flow and oxygenation [3,5] and have been used in a variety of sports science studies to examine oxygen dynamics during endurance and strength performance [4]. Their utilization provides valuable insight into the energetic demands of the muscle during exercise and recovery. NIRS devices may also provide real-time biofeedback on muscle oxygenation loss to ensure that each set of resistance exercise achieves a desired magnitude and duration of metabolic stress. Alternatively, the technology may be useful for fatigue management and allow for proper recovery between sets by ensuring that each set of resistance exercise is initiated following a certain level of muscle oxygenation recovery [6].

Recent interest has led to a variety of studies using NIRS to examine muscle oxygen dynamics during resistance exercise [4]. Yet information describing the changes in muscle oxygen saturation during dynamic resistance exercises across multiple sets is limited. Although resistance exercise is largely dependent on the anaerobic phosphagen system for energy production, increased flow of oxygen-rich blood to active musculature might facilitate longer duration efforts [1] and phosphocreatine resynthesis during recovery [7]. Indeed, muscle oxygenation has been shown to significantly decrease during the contraction phase and increase during rest intervals of resistance exercise protocols performed at different training loads [8,9,10], with different exercises (e.g., back squat versus front squat) [11,12], in younger versus older men [13], and differentially within the vastus lateralis and rectus femoris [10]. However, most studies are limited by small sample sizes, and no study has sought to describe muscle oxygenation dynamics during upper-body, multi-joint exercise. A better understanding of muscle oxygenation parameters during resistance exercise may provide insights into mechanisms that influence performance and recovery.

In addition to the standard resistance exercise performance metrics (e.g., repetitions and volume-load completed), changes have been noted in peak muscle oxygen saturation, the percentage change in muscle oxygenation, and the rate of muscle oxygen re-saturation during resistance exercise [8,9,10,11,12,13]. Among various factors, the influence of marginal control over pertinent participant characteristics relevant to the study’s dependent variables has been left unexplored. For instance, sex, resistance training experience, body mass, and strength can influence resistance exercise performance. These, along with an individual’s hemodynamic characteristics (i.e., heart rate and blood pressure), might directly or indirectly affect oxygen delivery during resistance exercise [14]. Therefore, the purpose of this study was to describe the physiological response of muscle oxygenation parameters during upper-body resistance exercise in recreationally resistance-trained men and women. Additionally, we sought to examine the differential effects of relevant participant characteristics on resistance training performance and muscle oxygen saturation dynamics. A better understanding of these variables may lead to programming strategies for improving responses to strength training and identify participant characteristics that may influence muscle oxygen saturation dynamics.

## 2. Materials and Methods

### 2.1. Experimental Design

A descriptive study design was employed to characterize the physiological response of muscle oxygenation parameters during upper-body resistance exercise in recreationally resistance-trained men and women. To increase sample size, data were collected from three research sites where the same standardized procedures using the same brand and model NIRS sensor were employed. The enrolled participants completed two sessions at their respective research laboratory separated by approximately 7 days. On the first session, 1-repetition maximum (1-RM) strength in the barbell bench press exercise was determined. Then, during the second session, participants completed a bench press protocol consisting of 5 sets at a load equal to 75% 1-RM performed to repetition maximum (RM). Prior to any session, participants were asked to avoid strenuous exercise and alcohol for 24 h and to arrive at the laboratory following an overnight fast (except water). Muscle oxygen saturation dynamics within the anterior deltoid were monitored for changes across all repetitions and rest periods of the bench press protocol.

### 2.2. Participants

The descriptive characteristics of the 61 recreationally resistance-trained men and women who volunteered to participate in this study are presented in Table 1. Participants’ height (±0.01 m) and weight (±0.1 kg) were recorded using a stadiometer and resting blood pressure and heart rate were recorded in duplicate and averaged using an automated blood pressure machine (Omron Healthcare Inc., 907XL Digital Blood Pressure Monitor; Hoffman Estates, IL, USA). To be considered recreationally resistance-trained, participants were required to have at least one year of resistance training experience that included the bench press exercise. Exclusion criteria included any serious physical limitations or illnesses that could potentially limit exercise performance, the ingestion of medications or performance-enhancing drugs that could affect performance or exercise assessment, and the inability to perform vigorous physical activity. Following a thorough explanation of the study design and procedures, each participant provided his or her written informed consent before participating in this study. This study was approved by the Institutional Review Boards at each university prior to participant recruitment and implementation.

### 2.3. Bench Press Strength Testing and Protocol

An RM strength test for the barbell bench press exercise was conducted using methods previously described [15]. Briefly, each participant performed a standardized warm-up consisting of dynamic stretches followed by two sub-maximal warm-up sets of 6–10 repetitions and 3–5 repetitions using a resistance approximately equal to 40–60% and 60–80% of the participant’s perceived 1-RM. Their 1-RM was then estimated by applying the Brzycki formula based on the number of repetitions performed to fatigue using a fixed weight [16]. Throughout the entire lift, participants were required to maintain contact between their feet and the floor, as well as their hips, shoulders, and head with the bench.

During the experimental trial, participants completed a bench-press-specific warm-up consisting of 8 repetitions at 40% 1-RM and 4 repetitions at 60% 1-RM separated by a two-minute rest period. Next, participants completed 5 bench press sets using a load equal to 75% 1-RM with 2 min of rest between each set. Each set was taken to volitional fatigue (i.e., RM) and was concluded when the participant could not complete another repetition without assistance or with proper technique. Proper technique was monitored and enforced during both sessions by a certified strength and conditioning specialist. Total repetitions and volume load (in kg; repetitions × load) completed were recorded on each set for analysis.

### 2.4. Muscle Oxygenation Assessment

Muscle oxygenation dynamics were measured with a portable NIRS sensor (Moxy, Fortiori Design, LLC, Hutchinson, MN, USA). This device is among the most common commercially available portable NIRS devices used for the evaluation of muscle oxygenation during strength training [2] and has shown to be valid and reliable for assessing muscle oxygenation parameters [5,17,18]. The device provides absolute concentration of oxygenated hemoglobin (HbO_2_), relative to total hemoglobin (tHb), output as muscle oxygen saturation [SmO_2_ = (HbO_2_/tHb) × 100)].

**Figure 1:**
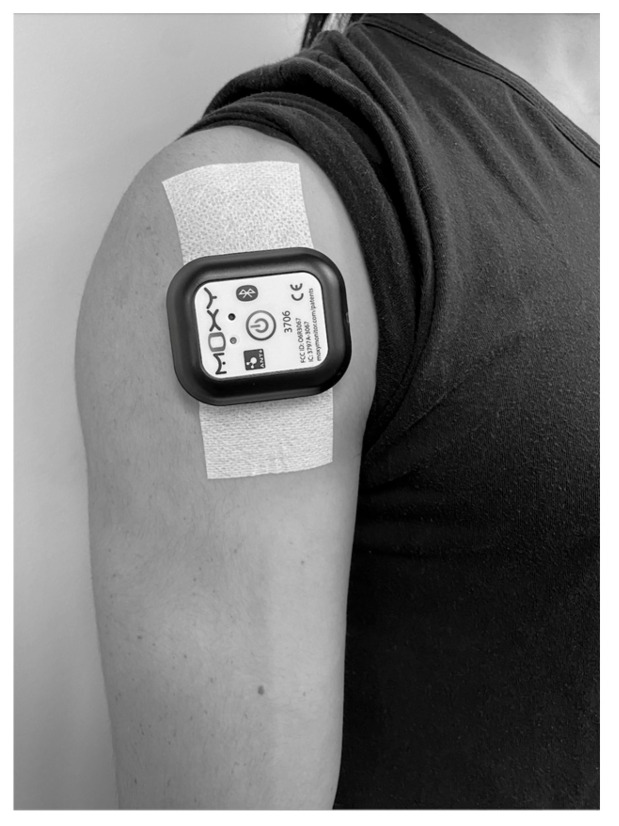
Moxy NIRS sensor attachment.

Prior to completing the bench press protocol, a Moxy NIRS sensor was attached to the anterior deltoid muscle belly at the midpoint between the clavicle and the insertion of the deltoid to the humerus (Figure 1) [19]. When necessary, excess hair was shaved, and the skin surface was swabbed with an alcohol wipe prior to attachment of the unit. A Moxy adhesive bandage was used to hold the device in place and shield it from external light. Consistent with prior research [20], it was determined through pilot testing that the anterior deltoid muscle allowed for best attachment to the skin for optimal sensor output, as opposed to the pectoralis major or pectoralis minor muscle.

Four variables were utilized to describe muscle oxygenation dynamics during the bench press sets and in recovery between sets. The first was the percent change in SmO_2_ (∆%SmO_2_) from the start (SmO_2_start) to the end (SmO_2_stop) of each set. SmO_2_start was recorded as the SmO_2_ value one second before each bench press set began, while SmO_2_stop was recorded as the SmO_2_ value at the end of the concentric phase of the final repetition of each set. The following formula was used to calculate Δ%SmO_2_ [9]:

(1) $$\Delta \%{\mathrm{SmO}}_{2}=\left(\left(\frac{{\mathrm{SmO}}_{2}\mathrm{stop}\;\times \;100}{{\mathrm{SmO}}_{2}\mathrm{start}}\right)-100\right)\times -1$$

The second variable used to analyze SmO_2_ values was muscle reoxygenation time (SmO_2_RecT). This value was represented by the amount of time it took for SmO_2_ to return to a baseline level (a value that stagnated for 5 s) following the end of each bench press set. The third value was the muscle oxygen re-saturation rate (SmO_2_RecSlope) and was measured as the slope of SmO_2_ values for 30 s immediately following the final repetition of each bench press set. The fourth variable, SmO_2_Peak, was recorded as the highest measured SmO_2_ value achieved during each recovery period between sets. Starting and ending SmO_2_ values, ∆%SmO_2_, SmO_2_RecSlope, and SmO_2_Peak were maintained for statistical analysis.

### 2.5. Statistical Analysis

The assumption of normality was initially verified via the Shapiro–Wilk test before separate, two-way (sex [men, women] × time [sets 1–5]) repeated measures analyses of variance (ANOVA) were performed on performance (repetitions and volume load completed) and muscle saturation variables. To examine the effect of relevant controlling variables (i.e., participant characteristics), separate analyses of covariance (ANCOVA) with repeated measures were performed on each performance and muscle saturation variable. All descriptive variables presented in Table 1 were individually assessed as covariates for each outcome variable. For all statistical analyses, a Greenhouse–Geisser correction was applied to degrees of freedom whenever the assumption of sphericity was violated. Following any significant F-ratio, pairwise comparisons were made between time points using the Bonferonni adjustment. All analyses were further assessed using effect sizes (η^2^_p_: Partial eta squared). Interpretations of effect size were evaluated [21] at the following levels: small effect (0.01–0.058), moderate effect (0.059–0.137), and large effect (>0.138). Significance was accepted at an alpha level of *p* ≤ 0.05. All performance data are reported as mean ± standard deviation (SD), while all data for comparisons amongst covariates are presented as mean ± standard error (SE).

## 3. Results

### 3.1. Bench Press Performance

Repeated measures ANOVA revealed main effects for sex (F = 5.3, *p* = 0.025, η^2^_p_ = 0.08) and sets (F = 289.7, *p* < 0.001, η^2^_p_ = 0.83) with repetitions completed. Women typically completed more repetitions than men, and fewer repetitions were completed on each successive set compared to its previous set. However, the sex × sets interaction noted for volume load (F = 37.9, *p* < 0.001, η^2^_p_ = 0.39) indicated that men completed more work than women, and that declines across successive sets occurred for both sexes up to set 3 (*p* < 0.05). From there, volume load further declined from sets 3 to 4 for men only (*p* = 0.013). Sex and set comparisons for bench press performance are presented in Table 2.

**Figure 2:**
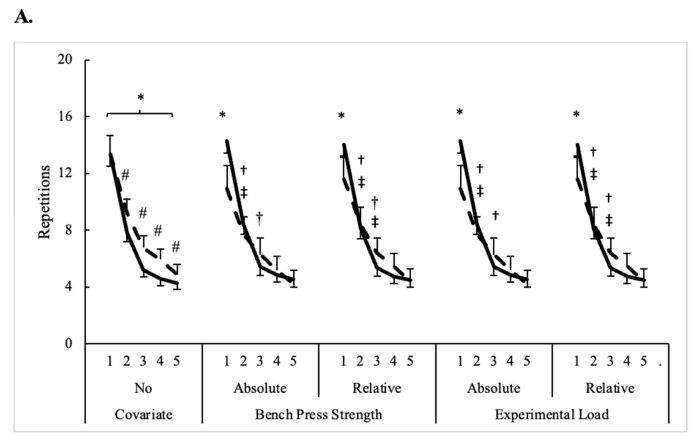

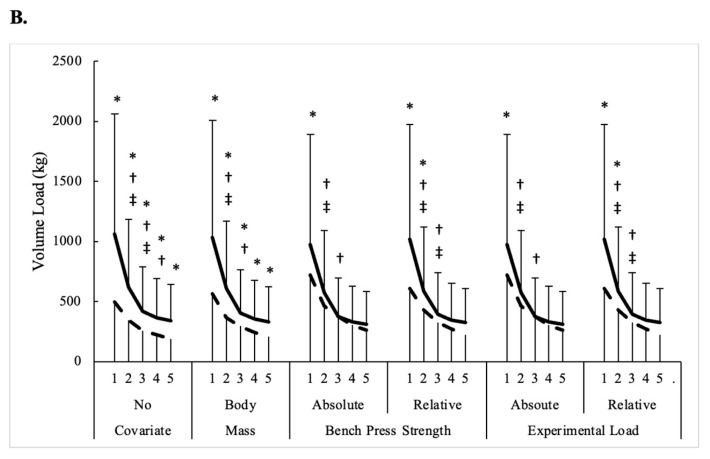
Influence of different covariates on (**A**) repetitions and (**B**) volume load completed across five sets of bench press in men and women (mean estimates ± SE). Solid line = men; Dashed line = women; * = Significant (*p* < 0.05) difference between men and women; † = Significant (*p* < 0.05) difference from previous set for men; ‡ = Significant (*p* < 0.05) difference from previous set for women; # = Significant (*p* < 0.05) difference from previous set in all participants.

The influence of different covariates on repetitions and volume load completed across five sets of bench press in men and women is illustrated in Figure 2. Bench press strength (absolute and relative) and the experimental load used on five sets of bench press (absolute and relative) were the only significant covariates (*p* < 0.01) for performance measures, and, in each instance, sex x time interactions (F = 6.1–14, *p* ≤ 0.002, η^2^_p_ = 0.10–0.19) were observed. Regardless of the covariate, the repetitions completed were only different between men and women on set 1. By contrast, men completed a higher volume load on each set only when body mass was equated at 84.0 kg. When absolute 1-RM strength (equated at 92.5 kg) and experimental load (equated at 69.4 kg) were the covariates, differences were only seen on set 1, whereas sets 1 and 2 were different when relative 1-RM (equated at 1.09 kg) and relative experimental load (equated at 0.82 kg) were the covariates. Differences between repetitions completed on consecutive sets were no longer present after set 3 in men and women when considering relative load covariates. Otherwise, with absolute load covariates, repetitions completed by women were consistent on consecutive sets after set 2. Except when body mass was the covariate, the set on which volume load became consistent mirrored what was observed for repetitions completed. When body mass was the covariate, volume load became consistent one set earlier for both men and women compared to what was revealed by repeated measures ANOVA.

### 3.2. Muscle Oxygenation

**Figure 3:**
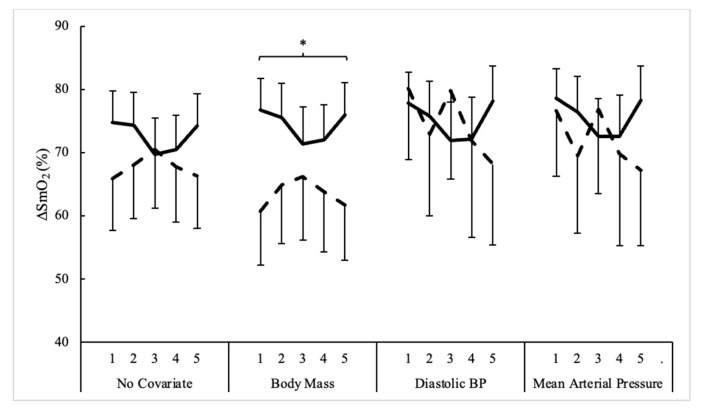
Influence of different covariates on the percent change in muscle oxygen across five sets of bench press in men and women (mean estimates ± SE). Solid line = men; Dashed line = women; ∆%SmO_2_ = loss of muscle oxygenation; * = Significant (*p* < 0.05) difference between men and women.

Sex and set comparisons for changes in muscle oxygenation measures during bench press exercise are presented in Table 2. According to repeated measures ANOVA, the main effects for sets occurred for SmO_2_RecSlope (F = 5.0, *p* = 0.001, η^2^_p_ = 0.09) and SmO_2_Peak (F = 7.0, *p* < 0.001, η^2^_p_ = 0.12). SmO_2_RecSlope significantly declined on sets 4 and 5 compared to set 1, while SmO_2_Peak was lowest on set 5 compared to all other sets. No differences were seen with ∆%SmO_2_ or SmO_2_RecT. Moreover, body mass (*p* = 0.013), diastolic blood pressure (*p* = 0.044), and mean arterial pressure (*p* = 0.033) for ∆%SmO_2_ were the only significant covariates noted amongst all muscle oxygenation variables. However, ANCOVA did not reveal any differences between sexes or across sets except for a main effect for sex when body mass was the covariate for ∆%SmO_2_ (F = 5.4, *p* = 0.024, η^2^_p_ = 0.09). Here, the percent change in muscle oxygen was generally greater in men compared to women when body mass was equated at 84.0 kg. The influence of different covariates on ∆%SmO_2_ across five sets of bench press in men and women is illustrated in Figure 3.

## 4. Discussion

There remains a need to better understand the utility and application of wearable technology such as NIRS devices for monitoring physiological parameters during resistance training. This study aimed to describe muscle oxygenation dynamics during upper-body resistance exercise in recreationally resistance-trained men and women. Additionally, this study aimed to identify relevant participant characteristics that may influence muscle oxygen saturation dynamics. As expected, repetitions and volume-load completed declined across five sets of bench press when not controlling for any characteristics, and performance was different between men and women. Then, when body mass, strength, and the experimental load were controlled, observed differences between sexes and across sets were not the same as without these controls. For muscle oxygenation, no differences were seen with ∆%SmO_2_ across sets. No sex differences and only a few set differences in muscle oxygen saturation dynamics were seen without employing any covariates (i.e., SmO_2_RecSlope and SmO_2_Peak), a sex difference appeared when controlling for body mass, and the previously observed set differences in SmO_2_RecSlope and SmO_2_Peak disappeared with each covariate. Body mass, diastolic blood pressure, and mean arterial pressure were identified as factors that could influence observed responses. These findings help describe the muscle oxygenation response during upper-body resistance training and demonstrate the importance of strict study controls for relevant participant characteristics when examining muscle oxygenation dynamics surrounding resistance exercise.

When engaging in resistance exercise, muscles require more oxygen due to the increased demand for energy, causing oxygen saturation in the muscle tissue to change. Previous studies show that muscle tissue oxygenation decreases during working sets and gradually returns to pre-exercise values during the rest interval between sets [8,9,10,11,12]. Yet few studies have explored the real-time changes in muscle oxygenation obtained by NIRS during dynamic resistance exercise in healthy adults. Hoffman et al. [8] reported similar muscle deoxygenation per set in the vastus lateralis muscle (~75%) during four sets of the squat exercise using either a low-intensity, high-volume (15 repetitions at 60% 1-RM) or high-intensity, low-volume (4 repetitions at 90% 1-RM) protocol. Davis et al. [11] measured the change in vastus lateralis muscle oxygenation during back and front squats using 3 sets of 15 repetitions at 70% 1-RM. While the ∆%SmO_2_ was not significantly different between back and front squats, the ∆%SmO_2_ did increase in the second (~55%) and third set (~56%) compared to the first set (~45%). Additionally, Gomez-Carmona et al. [9] compared several different back-squat training protocols and showed that volume, intensity, and level of effort influenced oxygenation of the vastus lateralis.

The only studies that have described muscle oxygenation in the deltoid muscle during a bench press exercise using NIRS (albeit with a different device: InSpectra^TM^ Tissue Oxygenation Monitor, Hutchinson Technology, Hutchinson, MN, USA) have been cross-over, placebo-controlled trials examining the impact of a dietary supplement [20,22]. Both studies had male participants perform 10 sets of bench press to muscular failure using a load equal to 50% of 1-RM. When examining the mean difference between SmO_2_start and SmO_2_stop values for each set during the placebo trials, Trepanowski et al. [22] reported ∆%SmO_2_ of approximately 35%, and Bloomer et al. [20] reported ∆%SmO_2_ of approximately 47%. Therefore, these studies reported lower ∆%SmO_2_ during each set as compared to those reported in our current study (~72.7%). However, similar to our findings, the changes in SmO_2_ did not significantly differ across the 10 sets. Therefore, it appears that the changes in muscle oxygenation remain relatively constant over the first 5–10 working sets of the bench press exercise using 50–70% 1-RM. It is also important to recognize that data provided from various NIRS devices available on the market can be complicated by different technology in the wearable devices.

In this current study, body mass, diastolic blood pressure, and mean arterial pressure were significant covariates for ∆%SmO_2_. This finding indicates that these participant characteristics may influence muscle oxygen saturation dynamics and may be important factors to control for in future studies. Even amongst individuals with resistance training experience, a wide range of body sizes, muscle masses, and fiber type distributions may be recruited for a study. While speculative, a greater body mass may make it more difficult for vasculature to supply exercising muscle with oxygen during training. While body mass and strength are naturally different between men and women, strength differences cease to exist when made relative to body mass [23,24,25]. Thus, body mass and relative strength can be appropriate covariates. Resting blood pressure influences circulation both at rest and during exercise and plays a crucial role in delivering oxygen-rich blood to active muscles [26]. Similarly, resting diastolic blood pressure and mean arterial pressure were identified as influential factors for future studies to consider and may have implications for exercise treatment studies in clinical populations. Muscle oxygenation may be impacted by factors related to blood pressure including activity of nitric-oxide-stimulating pathways, which play a critical role in endothelial function, promoting the relaxation of vascular smooth muscle and subsequent dilation, which may favorably impact blood flow to the working muscles [27].

This study does have some limitations worth noting. While the multi-site analysis allowed for an increased sample size, three different portable NIRS sensors were used for data collection. However, all processes were performed following the manufacturer’s recommendations with the same analysis procedures. Next, the findings from this study are specific to the bench press exercise with muscle oxygen saturation assessment of the anterior deltoid muscle and cannot be extrapolated to other prime mover muscles such as the pectoralis major. This study also included a relatively homogenous group of recreationally resistance-trained men and women; therefore, the results cannot be extrapolated to untrained individuals or elite athletes. Moreover, NIRS does not directly measure blood flow in the same manner as flow-mediated dilation via ultrasound technology; therefore, caution must be taken when inferring blood flow from NIRS assessment.

## 5. Conclusions

Muscle oxygenation could be considered as a useful method for understanding skeletal muscle fatigue during resistance training. The findings of this study describe the muscle oxygenation response of the anterior deltoid muscle during the bench press exercise. Overall, when participants completed five bench press sets using a load equal to 75% 1-RM with 2 min of rest between each set, no sex differences and only a few set differences in muscle oxygen saturation dynamics were seen without employing any covariates. Similar muscle oxygen responses occurred during each set with differences limited to SmO_2_RecSlope and SmO_2_Peak during the fourth and fifth set. Body mass, diastolic blood pressure, and mean arterial pressure were identified as factors that could influence observed responses. These findings demonstrate the importance of strict study controls for relevant participant characteristics when examining muscle oxygenation dynamics surrounding resistance exercise.

## Figures and Tables

**Table 1:** Participant characteristics in controlling variables.

	Men (n = 44)	Women (n = 17)	All Participants (n = 61)
Age (y)	21.8 ± 2.6	20.2 ± 1.8	22.1 ± 0.4
Height (cm)	179 ± 6	166 ± 9	176 ± 1
Body mass (kg)	89.3 ± 15.7	70.3 ± 14.8	81.4 ± 1.7
Resistance Training Experience (y)	5.8 ± 3.1	3.9 ± 1.8	5.5 ± 0.5
Resting Blood Pressure (mmHg)			
Systolic	123 ± 9	115 ± 11	122 ± 2
Diastolic	66 ± 7	73 ± 7	67 ± 1
Mean Arterial	85 ± 7	87 ± 7	85 ± 1
Resting Heart Rate (bpm)			
Average	70 ± 11	65 ± 7	69 ± 2
Reserve	128 ± 11	134 ± 7	129 ± 2
Bench Press Strength (kg)			
Absolute	108.4 ± 25.6	51.5 ± 16.3	96.5 ± 4.2
Relative (per kg of body mass)	1.22 ± 0.25	0.75 ± 0.24	1.18 ± 0.04
Experimental Load (kg)			
Absolute	81.3 ± 19.2	38.6 ± 12.2	72.4 ± 3.1
Relative (per kg of body mass)	0.92 ± 0.18	0.56 ± 0.18	0.88 ± 0.03

**Table 2:** Performance and muscle oxygenation across five sets of bench press (mean ± SD).

	Set 1	Set 2	Set 3	Set 4	Set 5	Across All Sets
Repetitions						
Men	13.4 ± 2.7	7.8 ± 2.2	5.2 ± 1.9	4.6 ± 1.5	4.3 ± 1.6	7.1 ± 3.8
Women	13.4 ± 3.1	9.2 ± 1.6	6.8 ± 1.2	5.9 ± 1.7	4.9 ± 1.4	8.0 ± 3.4 *
All	13.4 ± 2.8	8.2 ± 2.1 #	5.7 ± 1.9 #	5.0 ± 1.6 #	4.4 ± 1.5 #	7.3 ± 3.7
Volume Load (kg)						
Men	1061 ± 230	617 ± 179 #	419 ± 175 #	363 ± 124 #	339 ± 129	560 ± 301
Women	497 ± 133 *	346 ± 96 *#	259 ± 83 * #	224 ± 81 *	187 ± 73 *	303 ± 124 *
All	904 ± 328	541 ± 201	375 ± 171	324 ± 129	297 ± 135	488 ± 251
∆%SmO_2_ (%)						
Men	74.7 ± 16.1	74.3 ± 18.5	69.7 ± 19.7	70.5 ± 19.6	74.2 ± 18.2	72.7 ± 2.4
Women	65.8 ± 18.1	68.1 ± 14.4	70.5 ± 17.6	67.7 ± 13.0	66.3 ± 13.3	67.7 ± 1.8
All	72.3 ± 17.0	72.6 ± 17.5	69.9 ± 19.0	69.8 ± 17.9	72.0 ± 17.3	71.3 ± 1.4
SmO_2_RecT (sec)						
Men	52.4 ± 14.2	51.7 ± 12.4	50.6 ± 12.4	54.3 ± 14.1	61.6 ± 19.0	54.1 ± 4.4
Women	55.4 ± 18.8	58.0 ± 24.6	60.1 ± 16.0	54.5 ± 15.7	55.1 ± 17.6	56.6 ± 2.4
All	53.4 ± 15.7	53.8 ± 17.3	53.7 ± 14.2	54.4 ± 14.5	59.4 ± 18.7	54.9 ± 2.5
SmO_2_RecSlope						
Men	1.12 ± 0.54	1.12 ± 0.57	0.98 ± 0.50	0.92 ± 0.53	0.80 ± 0.57	0.99 ± 0.14
Women	1.00 ± 0.69	0.82 ± 0.52	0.75 ± 0.53	0.74 ± 0.50	0.77 ± 0.55	0.82 ± 0.11
All	1.08 ± 0.59	1.02 ± 0.57	0.91 ± 0.51	0.86 ± 0.52 #	0.79 ± 0.56 #	0.93 ± 0.12
SmO_2_Peak (%)						
Men	80.7 ± 12.1	79.9 ± 11.9	79.3 ± 12.1	80.8 ± 9.4	77.7 ± 12.3	79.7 ± 1.3
Women	81.3 ± 11.8	80.8 ± 10.9	80.8 ± 11.8	79.4 ± 11.8	76.3 ± 14.1	79.7 ± 2.0
All	80.9 ± 11.9	80.2 ± 11.5	79.8 ± 11.9	80.3 ± 10.2	77.2 ± 12.8 #	79.7 ± 1.4

SmO_2_ = skeletal muscle oxygenation; ∆%SmO_2_ = loss of muscle oxygenation; SmO_2_RecT = muscle reoxygenation time; SmO_2_RecSlope = muscle oxygen re-saturation rate; SmO_2_Peak = highest SmO_2_ value achieved in rest period; * = Significant (*p* < 0.05) difference between men and women; # = Significant (*p* < 0.05) difference from previous set.

## Data Availability

Data are contained within the article.

## References

[1] Tuesta M., Yáñez-Sepúlveda R., Verdugo-Marchese H., Mateluna C., Alvear-Ordenes I. (2022). Near-infrared spectroscopy used to assess physiological muscle adaptations in exercise clinical trials: A systematic review. Biology.

[2] Miranda-Fuentes C., Chirosa-Ríos L.J., Guisado-Requena I.M., Delgado-Floody P., Jerez-Mayorga D. (2021). Changes in muscle oxygen saturation measured using wireless near-infrared spectroscopy in resistance training: A systematic review. Int. J. Environ. Res. Public Health.

[3] Grassi B., Quaresima V. (2016). Near-infrared spectroscopy and skeletal muscle oxidative function in vivo in health and disease: A review from an exercise physiology perspective. J. Biomed. Opt..

[4] Perrey S., Ferrari M. (2018). Muscle oximetry in sports science: A systematic review. Sports Med..

[5] Crum E., O’connor W., Van Loo L., Valckx M., Stannard S. (2017). Validity and reliability of the Moxy oxygen monitor during incremental cycling exercise. Eur. J. Sport Sci..

[6] Spiering B.A., Clark B.C., Schoenfeld B.J., Foulis S.A., Pasiakos S.M. (2022). Maximizing Strength: The Stimuli and Mediators of Strength Gains and Their Application to Training and Rehabilitation. J. Strength Cond. Res..

[7] McCully K., Iotti S., Kendrick K., Wang Z., Posner J., Leigh J., Chance B. (1994). Simultaneous in vivo measurements of HbO2 saturation and PCr kinetics after exercise in normal humans. J. Appl. Physiol..

[8] Hoffman J.R., Im J., Rundell K.W., Kang J., Nioka S., Speiring B.A., Kime R., Chance B. (2003). Effect of muscle oxygenation during resistance exercise on anabolic hormone response. Med. Sci. Sports Exerc..

[9] Gómez-Carmona C.D., Bastida-Castillo A., Rojas-Valverde D., de la Cruz Sánchez E., García-Rubio J., Ibáñez S.J., Pino-Ortega J. (2020). Lower-limb dynamics of muscle oxygen saturation during the back-squat exercise: Effects of training load and effort level. J. Strength Cond. Res..

[10] Azuma K., Homma S., Kagaya A. (2000). Oxygen supply-consumption balance in the thigh muscles during exhausting knee-extension exercise. J. Biomed. Opt..

[11] Davis P.R., Yakel J.P., Anderson D.J. (2020). Muscle oxygen demands of the vastus lateralis in back and front squats. Int. J. Exerc. Sci..

[12] Timón R., Ponce-González J.G., González-Montesinos J.L., Olcina G., Pérez-Pérez A., Castro-Piñero J. (2017). Inertial flywheel resistance training and muscle oxygen saturation. J. Sports Med. Phys. Fit..

[13] Gepner Y., Wells A.J., Gordon J.A., Arroyo E., Varanoske A.N., Coker N.A., Fukuda D.H., Stout J.R., Hoffman J.R. (2019). Differences in muscle oxygenation between young and middle-aged recreationally active men during high-volume resistance exercise. Kinesiology.

[14] Dipla K., Triantafyllou A., Koletsos N., Papadopoulos S., Sachpekidis V., Vrabas I.S., Gkaliagkousi E., Zafeiridis A., Douma S. (2017). Impaired muscle oxygenation and elevated exercise blood pressure in hypertensive patients: Links with vascular stiffness. Hypertension.

[15] Hoffman J.R. (2006). Norms for Fitness, Performance, and Health.

[16] Brzycki M. (1993). Strength testing—Predicting a one-rep max from reps-to-fatigue. J. Phys. Educ. Recreat. Danc..

[17] McManus C.J., Collison J., Cooper C.E. (2018). Performance comparison of the MOXY and PortaMon near-infrared spectroscopy muscle oximeters at rest and during exercise. J. Biomed. Opt..

[18] Feldmann A., Schmitz R., Erlacher D. (2019). Near-infrared spectroscopy-derived muscle oxygen saturation on a 0% to 100% scale: Reliability and validity of the Moxy Monitor. J. Biomed. Opt..

[19] Haynes J.T., Townsend J.R., Aziz M.A., Jones M.D., Littlefield L.A., Ruiz M.D., Johnson K.D., Gonzalez A.M. (2021). Impact of Red Spinach Extract Supplementation on Bench Press Performance, Muscle Oxygenation, and Cognitive Function in Resistance-Trained Males. Sports.

[20] Bloomer R.J., Farney T.M., Trepanowski J.F., McCarthy C.G., Canale R.E., Schilling B.K. (2010). Comparison of pre-workout nitric oxide stimulating dietary supplements on skeletal muscle oxygen saturation, blood nitrate/nitrite, lipid peroxidation, and upper body exercise performance in resistance trained men. J. Int. Soc. Sports Nutr..

[21] Cohen J. (1992). Statistical power analysis. Curr. Dir. Psychol. Sci..

[22] Trepanowski J.F., Farney T.M., McCarthy C.G., Schilling B.K., Craig S.A., Bloomer R.J. (2011). The effects of chronic betaine supplementation on exercise performance, skeletal muscle oxygen saturation and associated biochemical parameters in resistance trained men. J. Strength Cond. Res..

[23] Frontera W.R., Hughes V.A., Lutz K.J., Evans W.J. (1991). A cross-sectional study of muscle strength and mass in 45-to 78-yr-old men and women. J. Appl. Physiol..

[24] Doherty T.J. (2001). The influence of aging and sex on skeletal muscle mass and strength. Curr. Opin. Clin. Nutr. Metab. Care.

[25] Bishop P., Cureton K., Collins M. (1987). Sex difference in muscular strength in equally-trained men and women. Ergonomics.

[26] Stewart K.J., Sung J., Silber H.A., Fleg J.L., Kelemen M.D., Turner K.L., Bacher A.C., Dobrosielski D.A., DeRegis J.R., Shapiro E.P. (2004). Exaggerated exercise blood pressure is related to impaired endothelial vasodilator function. Am. J. Hypertens..

[27] Bailey S.J., Vanhatalo A., Winyard P.G., Jones A.M. (2012). The nitrate-nitrite-nitric oxide pathway: Its role in human exercise physiology. Eur. J. Sport Sci..

